# Spermine driven water deficit tolerance in early growth phases of sweet corn genotypes under hydroponic cultivation

**DOI:** 10.1038/s41598-025-86083-y

**Published:** 2025-01-13

**Authors:** Tahoora Batool Zargar, Mawia Sobh, Oqba Basal, Tibor Janda, Magda Pál, Szilvia Veres

**Affiliations:** 1 Department of Applied Plant Biology, Faculty of Agricultural and Food Sciences and Environmental Management, University of Debrecen, Debrecen, Hungary; 2 Department of Plant Physiology and Metabolomics, Agricultural Institute, Centre for Agricultural Research HUN-REN, Martonvásár, Hungary

**Keywords:** Polyamines, Bio-stimulants, Antioxidants, Drought, Corn, Plant physiology, Plant stress responses

## Abstract

Sweet corn is highly susceptible to water deprivation, making it crucial to identify effective strategies for enhancing its tolerance to water deficit conditions. This study investigates the novel application of Spermine as a bio-stimulant to improve sweet corn (*Zea mays* L. var. *saccharata*) resilience under hydroponic water deficit conditions. Four genotypes (Dessert, Messenger, Tyson, and Royalty) were treated with Spermine (0.2 mM foliar application), polyethylene glycol 6000 (8% and 12%), and their combinations. The impacts on growth parameters, photosynthetic performance, and oxidative stress markers were evaluated. Spermine significantly enhanced biomass parameters, counteracting the severe reductions caused by PEG-induced water deprivation. In the Dessert and Tyson genotypes, total biomass increased by 145%, while it increased by 118% in Messenger and 110% in Royalty when treated with Spermine under severe water deprivation. However, Spermine treatment application did not recorded higher differences compared to control under non water deficit conditions. In the Dessert genotype, root length increased by 36.6% under combined treatment compared to 12% PEG alone. Spermine also mitigated reductions in shoot length, improved by 90.6% and specific leaf area, with a notable 272.6% increase in Tyson under severe water deficit. Photosynthetic performance, including chlorophyll and carotenoid levels, was enhanced, with a 103.1% increase in relative chlorophyll content in Dessert under severe water deprivation. Spermine also reduced oxidative damage, as indicated by a 48.7% decrease in malondialdehyde levels in Tyson, and increased peroxidase activity, enhancing antioxidant defense in Messenger under severe water deprivation. The quantum efficiency of Photosystem II, which was significantly reduced by water deficit, showed substantial improvement with Spermine treatment, with increases of 107.2% in Tyson and 99.4% in Royalty under moderate water deprivation. These results highlight the potential of Spermine as an effective strategy to improve sweet corn resilience under water-limited conditions, offering a novel approach for sustainable crop management.

## Introduction

Sweet maize (*Zea mays* L. var. *saccharata*) represents an important agricultural crop, holding significant nutritional value and economic relevance globally. Sweet corn is well known for its sugary flavor and tender texture, rich in essential nutrients, including vitamins A and C, dietary fiber, and antioxidants such as lutein and zeaxanthin, which play an important role in maintaining eye health^1^. In agricultural sectors, sweet corn has a significant impact on both processed and fresh markets. Like dent corn, sweet corn hasn’t been widely developed for its ability to withstand water deprivation and exhibits limited genetic diversity to drought resistance. Despite being characterized by higher sugar content and unique starch biosynthesis, sweet corn cultivation faces considerable challenges as of changing climate and increasing drought prone agricultural lands^1^. Drought severely impacts sweet corn physiology, reducing water uptake, lowering seed moisture levels, and hindering germination. It stunts seedling growth, weakens vigor, and decreases biomass and yield. These effects intensify during early development, where limited water disrupts vital physiological processes critical for growth and establishment^2^. Decrease in production presents a significant thread to economic stability and food security.

In Hungary, particularly in the Great Hungarian Plain, sweet corn plays an important role in vegetable production but crop faces challenges from insufficient and unpredictable rainfall patterns^3^. Irrigation systems, although employed to mitigate the effects of drought, increase production costs and exacerbate sustainability challenges associated with water resource management^3^. As a result, novel approaches are essential to enhance the resilience of sweet corn under conditions of water deficit.

Recent research has suggested bio-stimulants, such as polyamines, as potent agents for improving plant resilience against abiotic stresses, particularly under water scarcity conditions^4^. Spermine (SPM), one of the most abundant polyamines, improves drought tolerance via various mechanisms, including the stabilization of cell membranes and the regulation of water transport through the modulation of aquaporin gene expression^5,6^. SPM improves stomatal conductance through the regulation of potassium channels and interaction with abscisic acid pathways, thereby increasing water use efficiency^7^. It also plays a crucial role in bolstering the antioxidant defense system by increasing the activity of key enzymes that counteract reactive oxygen species (ROS) generated during stress, thereby mitigating oxidative damage and maintaining cellular homeostasis^8,9^. The application of polyamines exogenously has been shown to elevate osmolyte levels, such as proline and soluble sugars, which contribute to osmotic adjustment and enhance stress tolerance in various plant species^10,11^.

While some studies have explored the foliar application of SPM in maize under drought stress^12,13^, there is a scarcity of research focusing specifically on sweet corn, especially within controlled hydroponic systems, underscoring the necessity of this study. This research aims to investigate the impact of foliar application of SPM on the morphological, physiological, and biochemical responses of four distinct sweet corn genotypes under simulated water deficit in a controlled hydroponic environment. The study focuses on understanding how these genotypes adapt to water deficit by examining specific goals: (1) morphological adaptations, including root volume and biomass variations; (2) physiological parameters, such as photosynthetic pigment levels, photosynthetic efficiency, and stomatal conductance; and (3) biochemical responses, including peroxidase antioxidant enzyme activity and lipid peroxidation levels. Detailed management strategies are being developed to incorporate SPM as a bio-stimulant alongside the selection of sweet corn genotypes tolerant to water deficits. This approach aims to enhance sustainable agricultural practices in response to the pressing challenge of water scarcity affecting sweet corn cultivation.

## Materials and methods

### Plant material and genotypes

In this experiment, four sweet corn genotypes (*Zea mays* var. *saccharata*) were considered: Dessert, Messenger, Tyson, and Royalty. All seeds were purchased (Seedplus Kft, Hungary). Messenger is a late-maturing variety, while Tyson and Royalty are mid-season, and Dessert varieties range from early to mid-early maturity^14–16^. In terms of primary use, Messenger and Dessert are versatile, suitable for both fresh consumption and processing, whereas Tyson is mainly geared towards fresh market sales. Each variety has distinct resistance features: Messenger excels in wind resistance (lodging), making it ideal for areas prone to heavy winds; Tyson offers broad disease resistance; Dessert varieties are adapted for early planting with strong resistance to multiple diseases; and Royalty is notable for its stress tolerance, particularly to environmental stresses such as heat and drought, and resistance to specific diseases like common rust, maize dwarf mosaic virus, and northern corn leaf blight. When it comes to kernel characteristics, Messenger features large, bright yellow, deep kernels arranged in straight rows with good tip fill, while Tyson and Dessert are appreciated for their sweet, smooth kernels, and Royalty produces more robust kernels with high yield stability. These genotypes were chosen for their diverse characteristics to provide a comprehensive evaluation of water deficit and treatment effects^14–16^.

### Seed preparation and germination

To prepare the seeds, they were first rinsed with tap water and then sterilized by soaking in a 6% H_2_O_2_ solution for 15 min. After sterilization, the seeds were thoroughly washed with distilled water, immersed in a 0.01 M CaSO_4_solution for 2 h, and rinsed again with distilled water. Uniform and healthy seeds were selected for germination, which occurred geotropically between moistened filter papers at 22 °C. Once seedlings reached the VE growth stage (The VE stage (Zadoks 09) marks seedling emergence, where the coleoptile becomes visible, and early photosynthesis begins. Root development accelerates, supporting water and nutrient uptake, making this stage critical for uniform growth and future development)^17^ and displayed good vigor, they were transplanted into 1.7 L plastic pots with four plants per pot set up in a hydroponic system having the nutritional solution composition indicated by Marschner et al.^18^ and replenished in every three days with fresh one.

### Experimental design

The experiment was designed to simulate water deprivation in a hydroponic system using polyethylene glycol (PEG 6000, VWR International BVBA Geldenaaksebaan, Leuven, Belgium). Six treatment groups were established: Control (ideal growth conditions with a foliar application of distilled water, at the same time and mode as it is described for SPM treatment), SPM (0.2 mM SPM), 8% PEG (water deprivation with 8% PEG), 12% PEG (water deprivation with 12% PEG), 8% PEG + SPM (water deprivation with 8% PEG and 0.2 mM SPM), and 12% PEG + SPM (water deprivation with 12% PEG and 0.2 mM SPM). Each treatment was replicated three times, resulting in a total of 72 pots (4 genotypes × 6 treatments × 3 replications). The concentrations were chosen based on earlier research defining PEG levels for simulating water deficits and the known effectiveness of 0.2 mM spermine in enhancing stress tolerance, allowing for assessment across a gradient of moderate to severe stress and under varying water conditions^19–23^.

### Water deprivation and treatment application

Two weeks after sowing, when plants reached the V4-V5 growth stage (4 to 5 established leaves, Zadoks scale 14–15), PEG was introduced into the hydroponic solution at concentrations of 8%, and 12% to simulate water deprivation. At the V6-V7 stage, plants were treated with a foliar application of 0.2 mM SPM (Sigma-Aldrich Kft., Budapest, Hungary) for seven consecutive days. The spray bottle was adjusted so that each spray released 1 mL of solution, with each plant receiving three sprays (totaling 3 mL per plant per application). After SPM treatment for one week at the V8-V9 vegetative stage (Zadoks scale 18–19), samples were taken to evaluate several morphological, physiological, and biochemical parameters.

### Data collection

1. **Morphological measurements**:Root and shoot lengths were measured with a ruler from the base of the plant to the tip of the root or shoot. Specific leaf area (SLA) was determined by drying five leaf discs per plant at 104.5 °C to a constant weight and then calculating SLA as the leaf area divided by the leaf dry weight^23^. Root volume was assessed using the water displacement method, based on Archimedes’ principle. The dry weight of roots and shoots were obtained after drying samples at 70 °C for four days to calculate total biomass.

2. **Physiological measurements**: Relative chlorophyll content was measured using a SPAD-502Plus device (Konica Minolta, Japan) on the last fully developed leaves. Photosynthetic pigments, including chlorophyll-a, chlorophyll-b, and carotenoids, were extracted using N, N-dimethylformamide and analyzed via UV-VIS spectrophotometry^24,25^. The HPLC method was used to analyze Lutein content in leaf extract using a Nucleosil C18 column and a UV/VIS detector (JASCO, Japan). Zeaxanthin was injected as a standard compound to identify peaks and calculate pigment contents^26^. Stomatal conductance was measured with an AP4 porometer (Delta-T, UK) on the youngest fully expanded leaves. Chlorophyll fluorescence parameters, such as maximum, minimal, and variable fluorescence, were evaluated using a PAM-2100 chlorophyll fluorometer (Walz GmbH, Effeltrich, Germany), with metrics including Fv/Fo, Fv/Fm, and ΔF/Fm’ were calculated^27^.

3. **Biochemical measurements**: By using trichloroacetic acid (TCA) as extraction buffer for leaf samples and measuring absorbance at 532 nm using a spectrophotometer (Metertech SP-830 PLUS, Taiwan) malondialdehyde (MDA) content was determined^28^. Peroxidase activity was assessed at the V8-V9 vegetative stage (Zadoks scale 18–19) by homogenizing leaf samples in 250 mM phosphate buffer (pH 6.8) and measuring absorbance at 460 nm in a reaction mixture containing sodium acetate buffer, hydrogen peroxide, and o-anisidine^29^.

### Statistical analysis

The experiment was set up in a completely randomized block design with three replications and four plants per replicate. Data was analyzed by two-way Analysis of Variance (ANOVA) to assess the effects of treatments and genotypes. To identify significant differences among treatments and genotypes Fisher’s Protected Least Significant Difference (LSD) test was done, with analyses performed using GenStat Release 12.1.

## Results

### Morphological traits

The application of spermine (SPM) and PEG-induced water deprivation significantly influenced root and shoot morphology across the four sweet corn genotypes (Dessert, Messenger, Tyson, and Royalty). Overall, SPM foliar spray positively affected root and shoot traits, while PEG treatments consistently led to notable reductions.

**Fig. 1:**
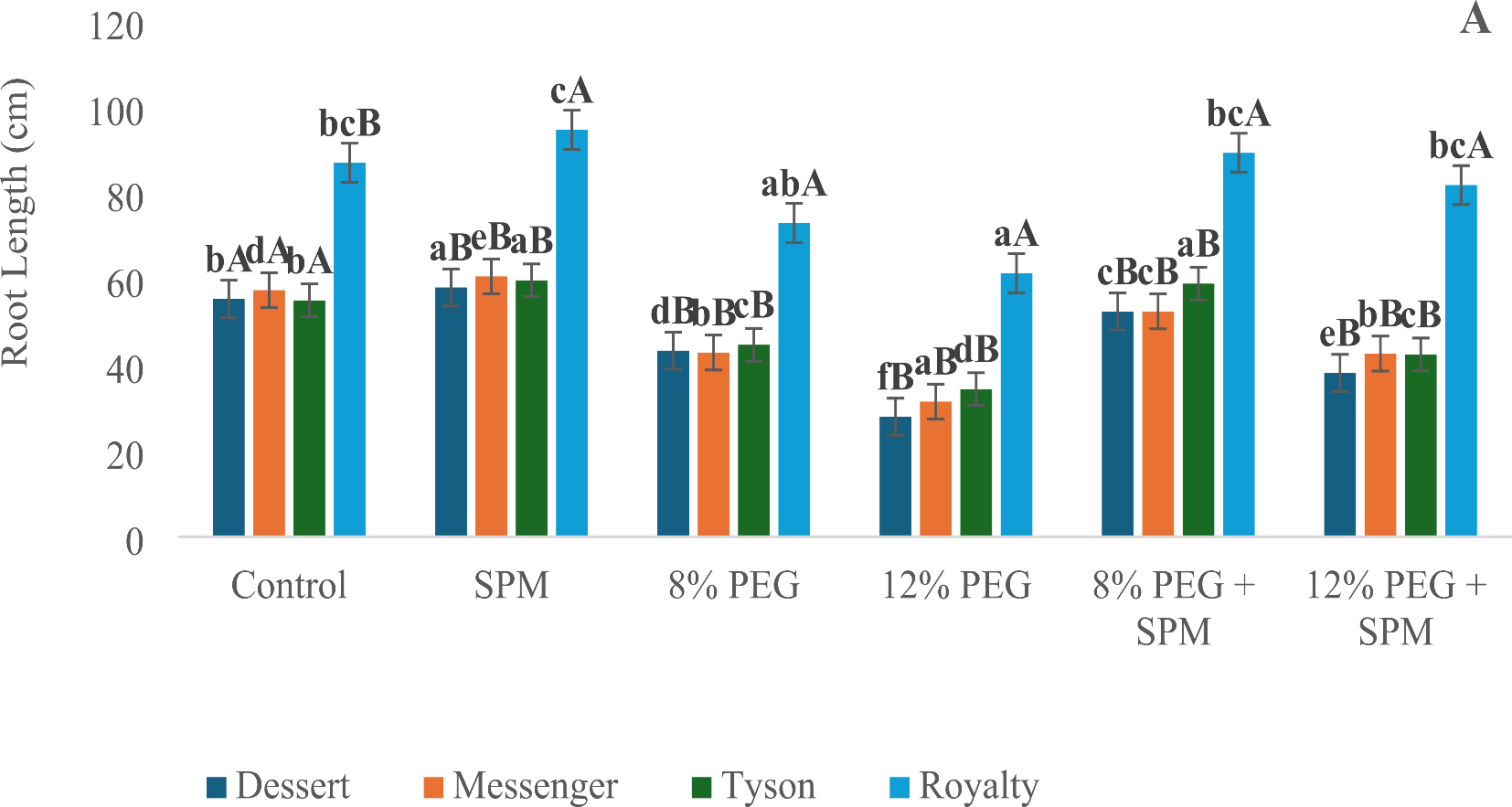
Effect of SPM and PEG-induced water deprivation on average root [**A**] and shoot [**B**] length (cm) on sweet corn genotypes (n = 3) with error bars indicating the standard error (s.e.m.). Different small letters indicate significant differences (*p* ≤ 0.05) among the treatments of each genotype, and different capital letters indicate significant differences (*p* ≤ 0.05) among genotypes within each treatment.

*Root length*: SPM application resulted in modest increases in root length, in all genotypes compared to control. In contrast, PEG treatments caused substantial declines, particularly under 12% PEG, which reduced root length by up to 49.6% in Dessert. However, the combination of SPM with PEG mitigated these adverse effects, leading to significant increase of 36.6%, 35.6%, 23.3%, 33.4% in Dessert, Messenger, Tyson and Royalty respectively under 12% PEG (Fig. 1A).

*Shoot length*: PEG treatments, especially at higher concentrations, significantly reduced shoot length, exemplified by a 50.6% reduction in Dessert, 59.2% in Tyson, 51.3% in Royalty under 12% PEG. The addition of SPM under PEG conditions effectively countered these declines, resulting in a remarkable 90.6% increase in shoot length in Tyson, 34.8% in Royalty, 28.6% in Dessert under severe water deprivation (Fig. 1B). At 8% PEG concentration SPM also increased shoot length by 39.6% and 32.2% in Messenger and Royalty respectively.

**Fig. 2:**
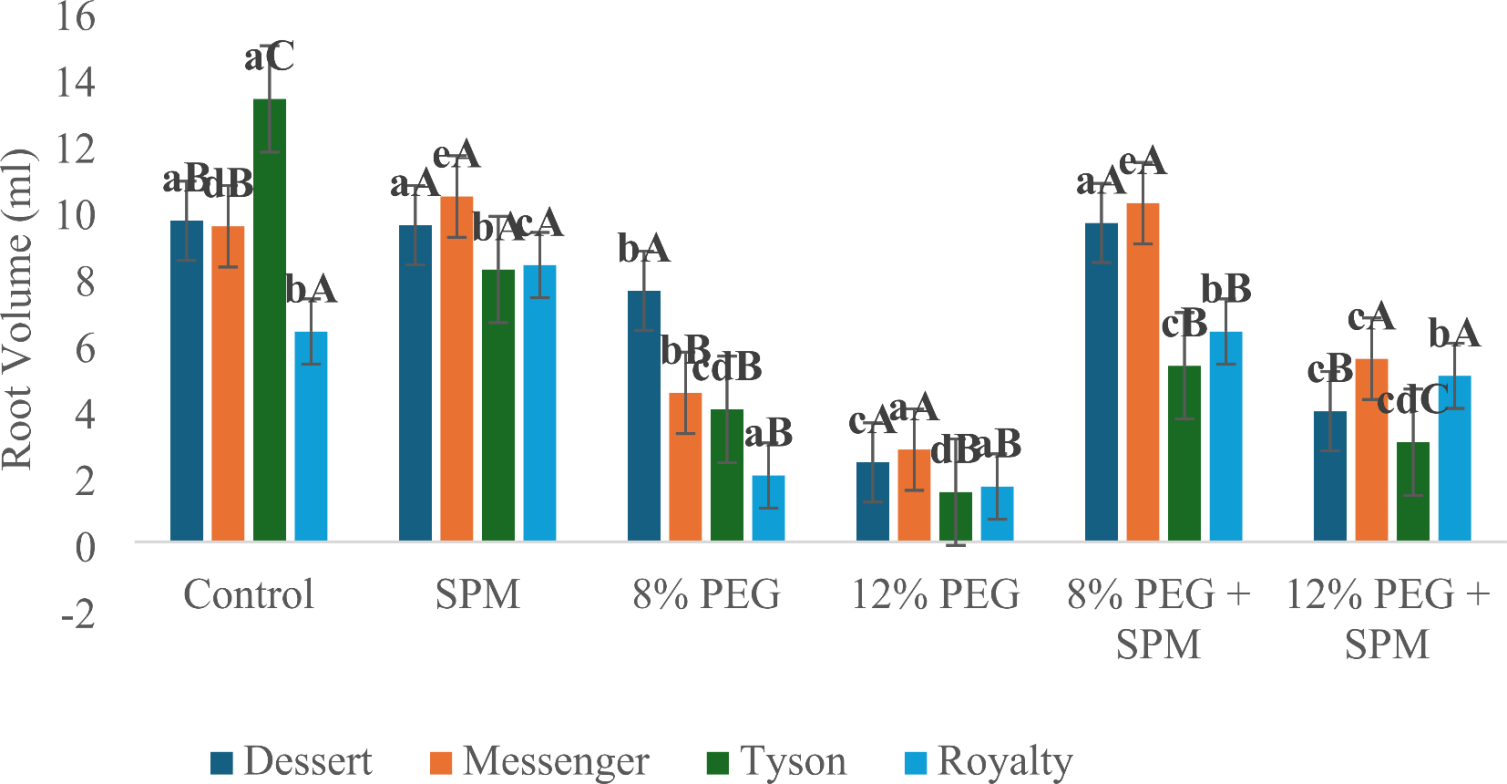
Effect of SPM and PEG-induced water deprivation on average root volume (cm^3^) on sweet corn genotypes (*n* = 3) with error bars indicating the standard error (s.e.m.). Different small letters indicate significant differences (*p* ≤ 0.05) among the treatments of each genotype, and different capital letters indicate significant differences (*p* ≤ 0.05) among genotypes within each treatment.

*Root volume*: Similar trends were observed in root volume, where PEG treatments alone caused significant reductions (up to 75% in Dessert under 12% PEG). SPM application under PEG conditions led to substantial improvements, including a 216.7% increase in Royalty under 8% PEG and under 12% PEG significant increase of 64.4%, 98.3%, 100.0% and 199.9% in Dessert, Messenger, Tyson and Royalty respectively was recorded on treatment with SPM (Fig. 2).

Total Biomass: The results show that treatment with SPM significantly increased biomass under PEG-induced water deprivation across all genotypes. Compared to 8% PEG, the 8% PEG + SPM treatment led to biomass increases of 134.5% in Dessert, 337.3% in Messenger, 134.5% in Tyson, and 86.2% in Royalty. Similarly, adding SPM to 12% PEG increased biomass by 145.3% in both Dessert and Tyson, 118.2% in Messenger, and 109.6% in Royalty (Table 1).

**Table 1:** Effect of SPM on total biomass (g) in water deprived sweet corn genotypes (n = 3). Different small letters indicate significant differences (*p* ≤ 0.05) among the treatments of each genotype, and different capital letters indicate significant differences (*p* ≤ 0.05) among genotypes within each treatment. *Specific Leaf Area (SLA)*: SPM had genotype-dependent effects on SLA under normal conditions, increasing SLA in dessert and Messenger while decreasing it in Tyson and Royalty. PEG-induced water deprivation significantly reduced SLA across all genotypes, with reductions of up to 73.8% in Tyson under 12% PEG. However, SPM application under PEG treatments effectively mitigated these reductions, resulting in substantial increases in SLA, particularly a 272.6% improvement in Tyson under 12% PEG (Table 2).

Trait (g)	Treatments	Genotypes			
Total dry biomass		Dessert	Messenger	Tyson	Royalty
	Control	5.54^bA^	3.64^cA^	5.54^aA^	6.63^aA^
	SPM	7.88^aA^	5.65^bA^	7.88^aA^	5.71^aA^
	8% PEG	3.13^cA^	1.69^eA^	3.13^bA^	2.17^cA^
	12% PEG	1.37^dA^	1.32^eA^	1.37^cA^	1.14^cA^
	8% PEG + SPM	7.34^aA^	7.39^aA^	7.34^abB^	4.04^bB^
	12% PEG + SPM	3.36^cA^	2.88^dAB^	3.36^bcC^	2.39^cBC^

**Table 2:** Effect of SPM on specific leaf area (cm² g⁻¹) in water deprived sweet corn genotypes (*n* = 3). Different small letters indicate significant differences (*p* ≤ 0.05) among the treatments of each genotype, and different capital letters indicate significant differences (*p* ≤ 0.05) among genotypes within each treatment.

Trait (cm² g⁻¹)	Treatments	Genotypes			
		Dessert	Messenger	Tyson	Royalty
SLA	Control	69.22^cA^	68.14^dA^	75.72^aA^	91.41^fA^
	SPM	82.14^dAB^	72.29^eB^	48.96^abC^	88.81^eA^
	8% PEG	58.77^bA^	52.53^bAB^	33.71^bC^	38.77^bBC^
	12% PEG	44.05^aA^	32.16^aB^	19.85^bC^	28.24^aB^
	8% PEG + SPM	100.90^eA^	59.08^cC^	64.99^aBC^	76.48^cB^
	12% PEG + SPM	81.60^dA^	88.01^fA^	73.96^aA^	81.90^dA^

### Physiological traits

**Fig. 3:**
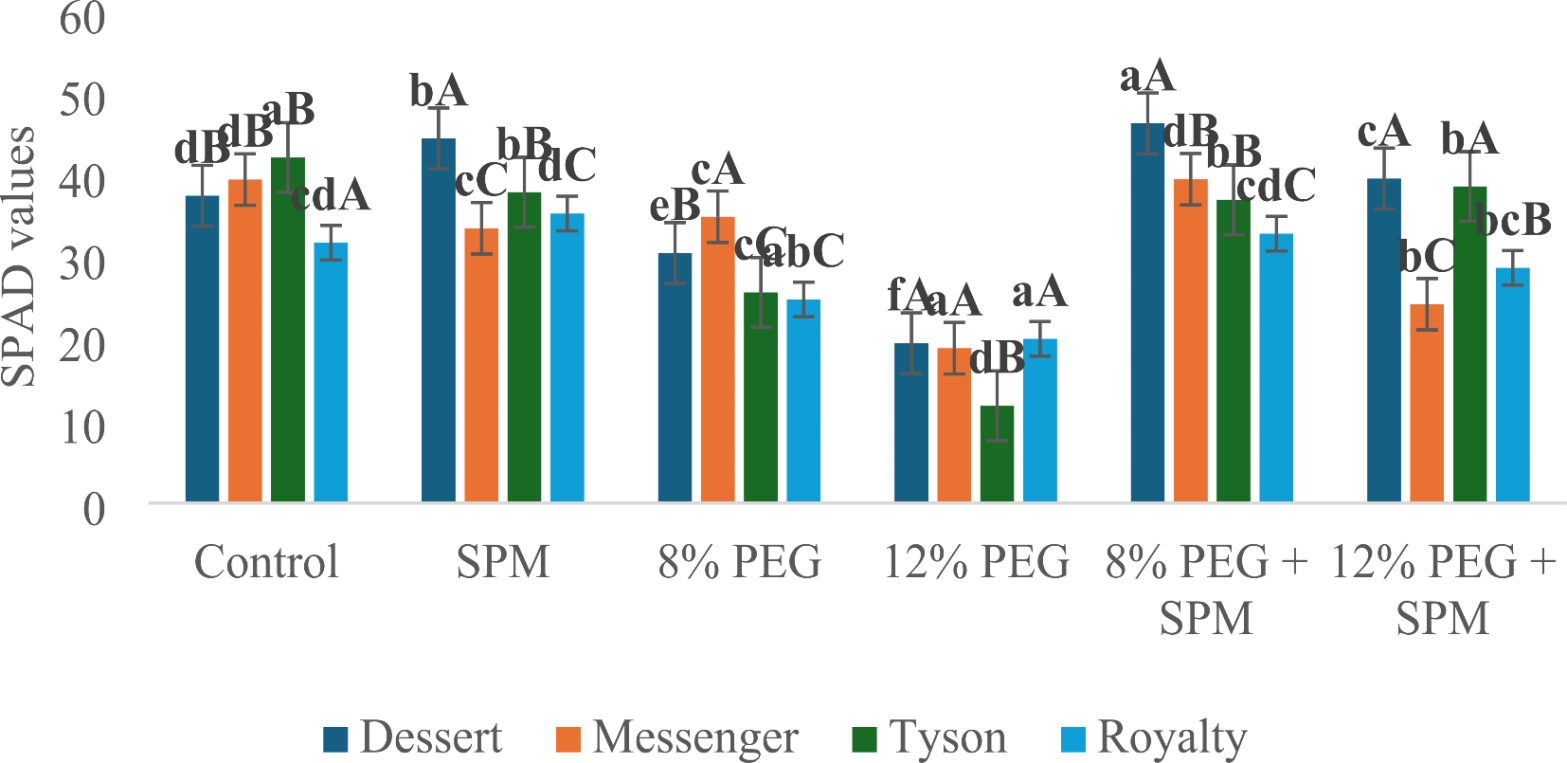
Effect of SPM on relative chlorophyll content in water deprived sweet corn genotypes (*n* = 3) with error bars indicating the standard error (s.e.m.). Different small letters indicate significant differences (*p* ≤ 0.05) among the treatments of each genotype, and different capital letters indicate significant differences (*p* ≤ 0.05) among genotypes within each treatment.

SPAD (relative chlorophyll content): SPM application generally enhanced SPAD values across genotypes, especially under PEG-induced water deprivation. In Dessert, SPAD increased by 18.7% with SPM alone and 103.1% to under 12% PEG when combined with SPM. Messenger exhibited a 28.4% increase in SPAD under severe water deprivation when SPM was applied. Tyson showed the most significant improvement, with SPAD values increasing by 225.4% under 12% PEG with SPM. Royalty also demonstrated a substantial increase by 43.3% under 12% PEG with SPM application (Fig. 3).

Photosynthetic pigments: SPM and PEG treatments significantly affected chlorophyll and carotenoid contents. In Dessert, SPM increased chlorophyll-a by 8.3% and total carotenoids by 13.5%, despite a reduction in chlorophyll-b. PEG treatments alone markedly reduced all pigments, but SPM addition under PEG conditions enhanced chlorophyll-a and carotenoids by up to 24.1% and 61.5% under 8% PEG in Dessert. Messenger showed a high increase in chlorophyll-b and carotenoids when SPM was combined with 12% PEG, with chlorophyll-b rising by 327.5%. Tyson benefited from SPM alone, showing increases in all pigments, and under PEG conditions, chlorophyll-b and carotenoids surged by 173.9% and 61.5%, respectively, with SPM under 12% PEG treatment. Royalty experienced significant increase in chlorophyll-a and carotenoids under PEG treatments with SPM, increasing by 48.4% and 118.2%, respectively under 12% PEG treatment (Table 3).

**Table 3:** Effect of SPM on photosynthetic pigments (mg g^−1^) in water deprived sweet corn genotypes (*n* = 3). Different small letters indicate significant differences (*p* ≤ 0.05) among the treatments of each genotype, and different capital letters indicate significant differences (*p* ≤ 0.05) among genotypes within each treatment. *Lutein content*: lutein content responded differently to SPM and PEG treatments across genotypes. In the dessert genotype, SPM reduced lutein by 25.0% compared to the control. However, under severe PEG conditions (12%), SPM led to a significant increase of 42.3%. In the Messenger genotype, SPM increased lutein by 41.1%, and combining SPM with moderate PEG (8%) led to the most substantial increase, doubling lutein content (102%) compared to PEG alone. For the Tyson genotype, while 12% PEG alone decreased lutein by 19.0%, SPM addition under moderate stress (8% PEG) resulted in a remarkable increase of 95.8%. In the Royalty genotype, SPM combined with 8% PEG led to a significant increase in lutein content by 102%, despite SPM alone having a minimal effect (Table 4).

Trait (mg g^−1^)	Treatment	Genotypes			
		Dessert	Messenger	Tyson	Royalty
Chlorophyll-a	Control	16.32^cB^	17.10^eB^	15.08^cA^	14.83^eA^
	SPM	17.68^dA^	16.21^dB^	17.22^dA^	13.47^dC^
	8% PEG	11.89^aB^	13.66^bA^	13.70^bA^	9.23^bC^
	12% PEG	10.79^aC^	12.04^aA^	11.17^aB^	7.85^aD^
	8% PEG + SPM	14.75^bA^	14.62^cA^	13.18^bA^	12.80^dA^
	12% PEG + SPM	13.86^bB^	15.92^dA^	13.97^bcB^	11.65^cC^
Chlorophyll-b	Control	6.70^cB^	7.83^bB^	4.35^cA^	6.82^bB^
	SPM	3.86^aB^	7.04^bA^	4.57^cB^	4.59^aB^
	8% PEG	5.60^abcA^	4.43^aA^	3.04^bB^	4.52^aA^
	12% PEG	4.44^abB^	2.70^aC^	1.85^aC^	7.75^bA^
	8% PEG + SPM	4.14^aA^	7.18^bA^	4.88^cA^	4.23^aA^
	12% PEG + SPM	5.90^bcB^	11.53^cA^	5.06^cB^	3.81^aC^
Total carotenoids	Control	5.42^dC^	4.07^bcB^	4.42^dB^	2.49^bA^
	SPM	6.15^eA^	4.60^dC^	5.42^eB^	3.72^dD^
	8% PEG	2.10^aC^	3.98^bA^	2.92^cB^	2.84^bcB^
	12% PEG	2.28^abB^	3.15^aA^	1.76^aC^	1.39^aD^
	8% PEG + SPM	3.39^cB^	4.44^cdA^	2.53^bC^	3.07^cBC^
	12% PEG + SPM	2.66^bC^	4.33^bcdA^	2.84^bcBC^	3.03^cB^

**Table 4:** Effect of SPM on lutein content (µg µl^−1^) in water deprived sweet corn genotypes (*n* = 3). Different small letters indicate significant differences (*p* ≤ 0.05) among the treatments of each genotype, and different capital letters indicate significant differences (*p* ≤ 0.05) among genotypes within each treatment. *Stomatal Conductance*: SPM generally enhanced stomatal conductance across all genotypes. Dessert recorded a 23.7% increase with SPM alone and a 59.7% recovery under 8% PEG with SPM. Messenger exhibited an 80.7% increase in stomatal conductance under 12% PEG with SPM. Tyson showed the highest response, with conductance increasing by 87.0% with SPM alone and further enhanced by 50.2% under 8% PEG. Royalty also demonstrated significant recovery, with stomatal conductance increasing by 44.2% under 12% PEG with SPM (Table 5).

Trait (µg µl^−1^)	Treatments	Genotypes			
Lutein		Dessert	Messenger	Tyson	Royalty
	Control	81.72^dB^	60.63^aA^	71.50^bAB^	85.86^eB^
	SPM	61.27^bC^	85.55^aAB^	90.22^aA^	72.25^bBC^
	8% PEG	93.25^eA^	70.10^aB^	65.19^cB^	75.01^cB^
	12% PEG	55.01^aB^	81.75^aA^	57.91^eB^	81.64^dA^
	8% PEG + SPM	101.86^fA^	71.53^aBC^	62.44^dC^	76.51^cB^
	12% PEG + SPM	78.27^cA^	58.83^aA^	50.10^fA^	59.26^aA^

**Table 5:** Effect of SPM on stomatal conductance (mmol H_2_O m^−2^ s^−1^) in water deprived sweet corn genotypes (*n* = 3). Different small letters indicate significant differences (*p* ≤ 0.05) among the treatments of each genotype, and different capital letters indicate significant differences (*p* ≤ 0.05) among genotypes within each treatment.

Trait (mmol H_2_O m^−2^ s^−1^)		Genotypes			
Stomatal Conductance		Dessert	Messenger	Tyson	Royalty
	Control	16.25^cB^	28.51^eC^	14.90^cB^	9.80^abcA^
	SPM	20.10^dB^	28.09^eA^	27.87^aA^	12.97^cC^
	8% PEG	14.20^bB^	20.81^cA^	14.63^cB^	8.17^abC^
	12% PEG	8.90^aA^	9.36^aA^	9.56^dA^	7.72^aB^
	8% PEG + SPM	22.68^eA^	25.58^dA^	21.97^bA^	11.13^bcB^
	12% PEG + SPM	13.88^bB^	16.91^bA^	12.40^cdBC^	11.13^bcC^

### Biochemical traits

**Fig. 4:**
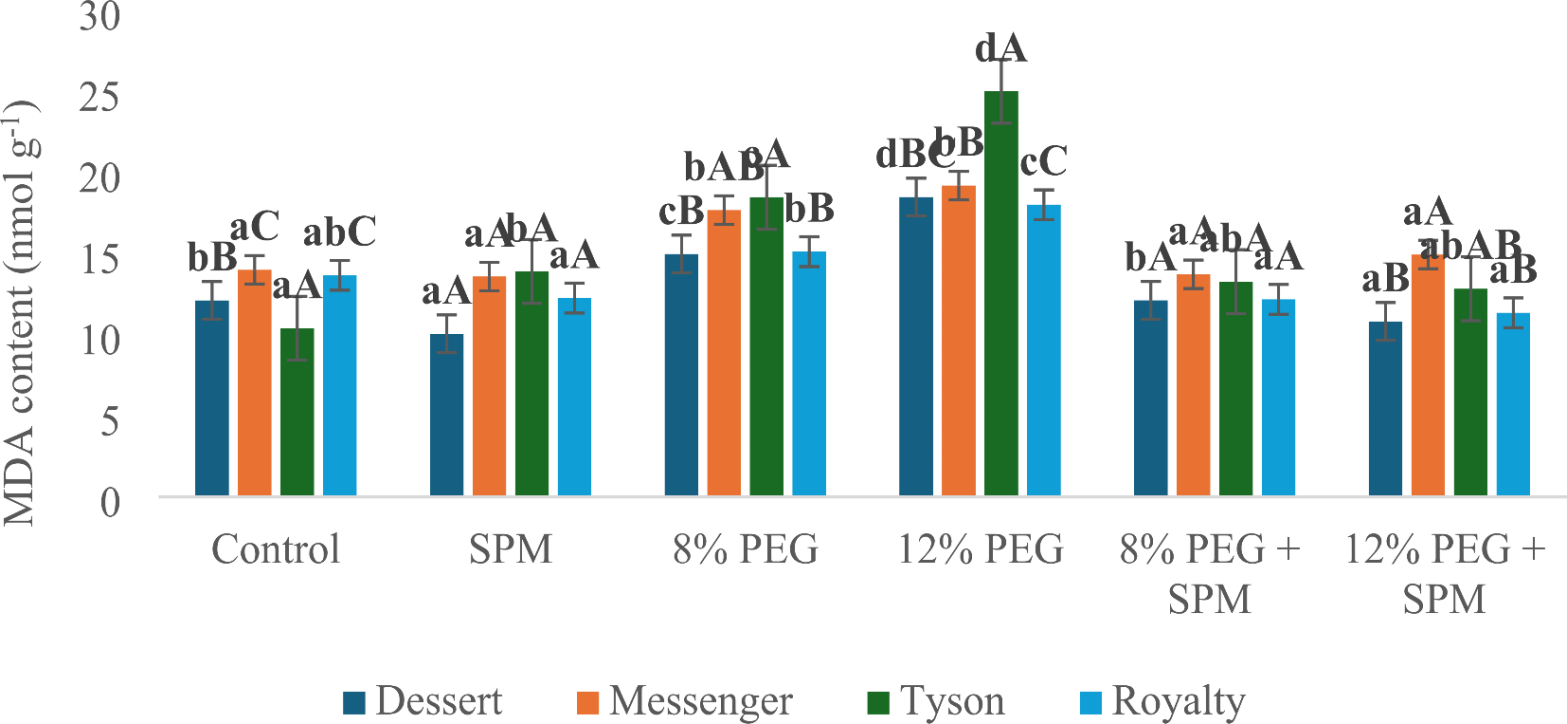
Effect of SPM on malondialdehyde content (nmol g^−1^) in water deprived sweet corn genotypes (*n* = 3) with error bars indicating the standard error (s.e.m.). Different small letters indicate significant differences (*p* ≤ 0.05) among the treatments of each genotype, and different capital letters indicate significant differences (*p* ≤ 0.05) among genotypes within each treatment.

Malondialdehyde (MDA) content: Malondialdehyde content, an indicator of lipid peroxidation, was generally reduced by individual treatment with SPM, except in Tyson where SPM alone increased MDA levels. In Dessert, SPM alone decreased MDA by a notable margin, and its combination with PEG resulted in reductions of up to 41.5% under 12% PEG. Messenger also showed reduced MDA levels by up to 22.1% with SPM under 12% PEG treatment. In Tyson, SPM application under PEG significantly lowered MDA by 48.7% under 12% PEG. Royalty exhibited a decrease in MDA by 19.4% and 37.0% under 8% and 12% PEG, respectively, when SPM was applied along with PEG (Fig. 4).

Peroxidase activity: SPM generally enhanced peroxidase activity, a crucial antioxidant enzyme, across all plants. In Dessert, SPM increased peroxidase activity by 100.8% under non water deficit conditions and further boosted it by 104.0% under 8% PEG. Messenger exhibited a substantial increase of 134.9% with SPM alone and up to 68.6% under 12% PEG when combined with SPM. In contrast, Tyson showed a decrease in peroxidase activity with SPM alone and under PEG treatments; however, the overall trend indicated reduced peroxidase activity. Royalty responded positively to SPM, with significant increases in peroxidase activity by 28.5% and 18.8% under 8% and 12% PEG, respectively (Table 6).

**Table 6:** Effect of SPM on peroxidase activity (µmol min^−1^ g^−1^) in water deprived sweet corn genotypes (*n* = 3). Different small letters indicate significant differences (*p* ≤ 0.05) among the treatments of each genotype, and different capital letters indicate significant differences (*p* ≤ 0.05) among genotypes within each treatment.

Trait (µmol min^−1^ g^−1^)	Treatments	Genotypes			
Peroxidase		Dessert	Messenger	Tyson	Royalty
	Control	10.83^aB^	8.33^aA^	21.39^bC^	25.01^cD^
	SPM	21.75^dB^	19.57^cC^	9.50^eD^	27.16^dA^
	8% PEG	10.92^aC^	17.60^cA^	15.82^cB^	16.28^aAB^
	12% PEG	14.61^bC^	13.46^bC^	29.64^aA^	20.95^bB^
	8% PEG + SPM	22.28^dB^	25.94^eA^	12.78^dC^	20.92^bB^
	12% PEG + SPM	19.62^cB^	22.69^dAB^	9.48^eC^	24.88^cA^

### Chlorophyll fluorescence parameters

Maximum Quantum Efficiency of Photosystem II (Fv/Fm): PEG treatments significantly decreased Fv/Fm ratios across all genotypes, indicating reduced photosynthetic efficiency. Dessert experienced a 10.7% decrease under 12% PEG, which was ameliorated by SPM, resulting in a 12.4% increase. Messenger recorded an 11.3% reduction under 12% PEG, partially improved by SPM with a 12.7% increase. Tyson exhibited the significant decline with a 21.6% reduction under 12% PEG, which was significantly countered by a 27.7% increase with SPM. Royalty also benefited from SPM under PEG, showing a 24.7% increase in Fv/Fm under 12% PEG.

Initial photochemical efficiency (Fv/F0): SPM positively affected Fv/F0 across most genotypes. Dessert showed a 4.8% increase with SPM alone and a 62.1% increase under 12% PEG with SPM. Messenger demonstrated a significant increase with a 96.9% increase under 8% PEG and a 61.8% increase under 12% PEG when SPM was applied. Tyson, despite a reduction with PEG alone, showed substantial improvement with SPM, increasing Fv/F0 by 248.9% under 8% PEG and 130.0% under 12% PEG. Royalty also exhibited substantial increases in Fv/F0 by 116.4% under 12% PEG with SPM.

*Quantum yield of PSII* (*ΔF/Fm’)*: Quantum yield was significantly influenced by both PEG and SPM treatments across all genotypes. In Dessert, SPM alone resulted in an increase in quantum yield, while PEG treatments reduced yield by up to 25.6% under 12% PEG. The combination of SPM with PEG led to substantial quantum yield improvements, with a 37.1% increase under 12% PEG. Messenger experienced slight yield decreases with SPM alone and PEG treatments, but SPM combined with PEG resulted in significant enhancements in quantum yield up to 96.7% under 8% PEG and 22.3% under 12% PEG. Tyson showed a decrease in yield with SPM alone and PEG treatments; however, SPM application under PEG conditions increased yield by 107.2% under 8% PEG and 43.2% under 12% PEG. In Royalty, SPM alone slightly increased quantum yield while 12% PEG treatment caused reductions of up to 29.6%. The application of SPM under PEG conditions resulted in significant increases of 99.4% under 8% PEG and 39.6% under 12% PEG (Table 7).

**Table 7:** Effect of SPM on chlorophyll fluorescence parameters (Fv/Fm, Fv/F0, ΔF/Fm’) in water deprived sweet corn genotypes (*n* = 3). Different small letters indicate significant differences (*p* ≤ 0.05) among the treatments of each genotype, and different capital letters indicate significant differences (*p* ≤ 0.05) among genotypes within each treatment.

Trait	Treatment	Genotypes			
		Dessert	Messenger	Tyson	Royalty
ΔF/Fm’	Control	0.75^cA^	0.75^cdA^	0.84^aA^	0.76^cA^
	SPM	0.76^cA^	0.74^cdA^	0.76^aA^	0.78^cA^
	8% PEG	0.66^bB^	0.70^bcA^	0.70^abA^	0.64^bB^
	12% PEG	0.56^aB^	0.63^aA^	0.56^bB^	0.53^aB^
	8% PEG + SPM	0.73^cA^	0.68^abAB^	0.75^aA^	0.63^bB^
	12% PEG + SPM	0.76^cA^	0.77^dA^	0.80^aA^	0.75^cA^
Fv/F0	Control	3.92^cA^	3.91^bcA^	3.63bA	4.19^cA^
	SPM	4.11^cA^	4.06^cA^	3.91^abA^	3.83^cA^
	8% PEG	3.18^bA^	2.96^abA^	1.71^cA^	2.81^bA^
	12% PEG	2.46^aA^	2.49^aA^	1.60^cB^	1.81^aB^
	8% PEG + SPM	4.09^cA^	2.87^abB^	4.27^aA^	2.00^aC^
	12% PEG + SPM	3.98^cA^	4.03^cA^	3.68^bA^	3.92^cA^
Fv/Fm	Control	0.80^cA^	0.80^aA^	0.78^aA^	0.81^cA^
	SPM	0.80^cA^	0.80^aA^	0.80^aA^	0.79^cA^
	8% PEG	0.76^bA^	0.73^aA^	0.62^bA^	0.74^bA^
	12% PEG	0.71^aA^	0.71^aA^	0.61^bB^	0.64^aB^
	8% PEG + SPM	0.80^cA^	0.74^aB^	0.81^aA^	0.66^aC^
	12% PEG + SPM	0.80^cA^	0.80^aA^	0.78^aA^	0.80^cA^

## Discussion

Water scarcity is a significant barrier to agricultural development, resulting in high concentrations of reactive oxygen species (ROS), such as superoxide and hydrogen peroxide, which adversely affect crop yields^30^. Sweet corn production faces considerable challenges due to the detrimental impacts of drought^31^. Water scarcity, exacerbated by climate change and erratic weather patterns, poses a serious threat to the growth and development of sweet corn plants. Prolonged periods of drought can severely hamper sweet corn productivity, as water constitutes a substantial portion of a plant’s biomass and is essential for various physiological processes, including growth and metabolism^32^.

Spermine and other polyamines present in plant cells can help plants withstand osmotic stress^33–35^. Polyamines, which are positively charged, can stabilize membranes under stress by attaching to negatively charged molecules such as proteins and phospholipids^36,37^. Spermine, which contains four nitrogen groups, buffers more efficiently than spermidine or putrescine^38^. In Arabidopsis mutants lacking spermine were more sensitive to osmotic stress, compared to wild-type plants^39^.

Our study demonstrates the potential of spermine in mitigating the adverse effects of water deficit on sweet corn genotypes by enhancing antioxidant defenses, supporting pigment stability, and improving photosynthetic efficiency and biomass accumulation under PEG-induced water deficit. The diverse responses recorded among the genotypes highlight the importance of genotype-specific mechanisms that interact with spermine to enhance tolerance.

Malondialdehyde (MDA) content and peroxidase (POD) activity are key aspects of the water deficit response, managing oxidative stress^28,29^. Osmotic stress results in an increased level of MDA, a major byproduct of lipid peroxidation known for extensive membrane damage, which increases permeability and compromises the function of membrane proteins and enzymes^40^. Decreased MDA content corresponds to increased POD activity, as enhanced POD activity can help detoxify reactive oxygen species (ROS), reducing lipid peroxidation and oxidative damage^41^. While all antioxidant enzymes are integral to protecting plants from oxidative damage, POD’s adaptability, broad functionality, and role in cell wall strengthening and lignin formation give it an edge in managing oxidative stress, especially under varied and prolonged environmental stresses. It acts as a versatile defender in the plant’s arsenal against ROS-induced damage^42^. Reduced levels of MDA directly correlate with increased POD activity in the genotypes Messenger, Dessert, and Royalty under severe water deprivation. Our results align with those of Da Silva et al.^43^, who reported that spermine increased POD activity under severe salt stress. Water deprivation increased MDA content in all four studied genotypes, indicating oxidative damage, with a significant increase at higher PEG concentrations. Our findings showed that spermine application notably reduced MDA levels under PEG-induced water deprivation, signifying a decrease in lipid peroxidation and oxidative damage. This aligns with a study in wheat where polyamine application reduced MDA levels under drought stress^4^and significantly reduced ROS levels compared to drought alone. In the genotype Messenger, this reduction in MDA was complemented by the highest increase in POD activity under spermine alone. The increase in POD activity suggests Messenger’s potential to prioritize biochemical defenses, limiting oxidative damage by efficiently detoxifying ROS, which in turn lowers MDA content. In contrast, in the genotype Tyson, POD activity decreased with spermine application under normal conditions, potentially explaining the unexpected increase in MDA in this context. This suggests a genotype-specific response, where POD activity in Tyson may only increase under stressful conditions. In Royalty, spermine boosted peroxidase activity under normal conditions however, during PEG treatments, it decreased. This decrease was counteracted by spermine application, suggesting that spermine plays a role in enhancing antioxidative defenses under stress^44^.

Significant variations in photosynthetic pigments were recorded among treatments of PEG and spermine in the Dessert, Messenger, Tyson, and Royalty genotypes. Reduction in oxidative damage via increased POD activity may have a direct effect on the stability of photosynthetic pigments, especially chlorophyll and carotenoids. Osmotic stress can significantly reduce all pigments due to increased chlorophyllase activity^45^and an imbalance in chlorophyll biosynthesis and degradation caused by excess ROS^46^. Genotypes with higher POD activity, such as Messenger, showed notable pigment stability under severe water deprivation with spermine. The high increase in chlorophyll-b content in Messenger with spermine under severe water deprivation may be linked to high POD activity and reduced oxidative damage indicated by lower MDA levels. In Messenger, spermine alone decreased chlorophyll-a and chlorophyll-b but increased total carotenoids, indicating a shift towards protective mechanisms. This increase in pigment content suggests the role of spermine in preserving the photosynthetic machinery, essential for light capture and energy conversion.

Likewise, in Dessert, along with enhanced POD activity, spermine treatment under high PEG concentration increased chlorophyll-a, total carotenoids, and chlorophyll-b content. In Tyson, water deprivation caused severe pigment reductions, but spermine mitigated these effects, especially with 12% PEG, resulting in significant increases in chlorophyll-b and total carotenoids, suggesting that spermine helps stabilize the photosynthetic machinery^43^. In Royalty, spermine alone decreased chlorophyll-a and chlorophyll-b but significantly increased total carotenoids, reflecting an adaptive response to enhance non-photochemical quenching and protect against photo-damage. PEG treatments alone led to declines in chlorophyll-a and had varied effects on chlorophyll-b and total carotenoids. However, spermine combined with PEG significantly increased chlorophyll-a and total carotenoids, particularly with 12% PEG, underscoring spermine’s stress-alleviating properties.

It is well-documented that polyethylene glycol (PEG), commonly used to induce water deficit conditions by generating osmotic stress, reduces chlorophyll content, indicating the adverse effects of osmotic stress. In our study, water deprivation resulted in a significant reduction in relative chlorophyll content in all four studied genotypes, aligning with previous findings. spermine application enhanced relative chlorophyll content under water deprivation. Our results suggest that spermine can partially or fully counteract the adverse effects of water deprivation on relative chlorophyll content, possibly by protecting the chloroplast structure^13,47^. The varied responses among genotypes suggest potential genotypic differences. For instance, the significant increase in SPAD in Tyson under higher PEG concentrations suggests that Tyson may possess unique mechanisms that enable better utilization of spermine for stress mitigation. Water deficit reduced chlorophyll content across all genotypes is consistent with findings in peanuts^48^. The improvement in photosynthetic pigments with spermine is attributed to its potential to protect the photosynthetic machinery, support cell division, and maintain the antioxidant system^4,43^, highlighting spermine’s crucial role in enhancing plant resilience to water deprivation.

Pigment stability and reduced oxidative damage can contribute to photosynthetic efficiency, as indicated by parameters like Fv/Fm, Fv/F0, ΔF/Fm’, and stomatal conductance. Genotypes Messenger and Tyson showed a significant increase in chlorophyll-b under 12% PEG upon application of spermine, and also recorded an increase in photosystem II efficiency parameters, including Fv/Fm, Fv/F0, and ΔF/Fm’. This indicates a direct link between pigment content and photosynthetic performance. However, stomatal conductance, Fv/Fm, Fv/F0, and ΔF/Fm’ consistently decreased across all genotypes under PEG-induced water deprivation. The reduction in Fv/Fm under water deprivation is primarily due to physical damage to thylakoid membranes, increased membrane permeability, oxidative stress, reduced ATP and NADPH production, and photoinhibition, which collectively impair the function of PSII and overall photochemical efficiency^49^. Nevertheless, spermine treatment mitigated these reductions. spermine improved and even increased Fv/Fm and Fv/F0 in the Dessert and Messenger genotypes, suggesting enhanced PSII efficiency and stress tolerance^44^. The increase in ΔF/Fm’ with spermine treatment is correlated with these improvements in photosynthetic parameters. As decreased photosynthetic activity under osmotic stress reduces quantum yield^40^, spermine treatment under water deprivation increased quantum yield in all genotypes, suggesting improved photosynthesis and PSII efficiency, leading to better physiological performance. For example, spermine treatment in the Tyson genotype resulted in significant improvements in both parameters and, consequently, in quantum yield, demonstrating a robust protective effect even under severe water deprivation-induced reductions in Fv/Fm and Fv/F0^50^.

A significant reduction in stomatal conductance at higher PEG concentrations aligns with previous findings in maize^51^, where decreased stomatal conductance was observed under drought conditions. This reduction could be attributed to decreased specific leaf area, reduced photosynthetic pigments, and impaired photosystem II efficiency. Under water deprivation, SLA also decreased across all studied genotypes, a common effect of water deficit that negatively impacts leaf growth^40^. However, the application of spermine increased both stomatal conductance and SLA under PEG treatments. Spermine’s protective potential aligns with previous studies indicating that polyamines improve cell growth^50^ and with research by Yousefi et al.^52^, which showed that exogenous polyamine application enhances photosynthetic efficiency under environmental stress. Heydari et al.^53^also reported that polyamines regulate photosynthesis quality, water use efficiency and net photosynthesis, enhancing plant resilience to water deficit by improving osmotic regulation, membrane stability and ROS scavenging. However, spermine reduced SLA in Tyson and Royalty under normal conditions, which may be explained by unique physiological differences across the genotypes^50^, though it also recorded the highest improvement with spermine application under water deprivation. These responses highlight the complexity of plant stress reactions and the promise of spermine in increasing stress tolerance.

Lutein is a crucial antioxidant, and spermine application increased its levels, further confirming its significance in stress reduction. Since lutein quenches ROS produced under stress, it is essential for safeguarding the photosynthetic machinery^41^. Particularly at lower PEG concentrations, the rise in lutein content after spermine treatment in certain genotypes, such as Dessert and Royalty, suggests an improved antioxidative defense system. Since greater lutein levels shield PSII from oxidative damage and ensure improved photosynthetic performance, this enhancement likely contributes to the observed increases in photosynthetic efficiency and quantum yield^54^. However, the decrease in lutein content in Royalty at higher PEG concentrations, even after spermine application, raises the possibility that high stress levels may overcome spermine’s protective benefits. This variation within each genotype suggests that the effectiveness of polyamines such as spermine is largely determined by genetic factors and innate stress tolerance mechanisms^55^.

The combined effects of reduced oxidative damage, stable pigments levels and maintained photosynthetic efficiency are reflected across the biomass production in genotypes. Spermine foliar application alleviated the adverse effects of PEG-induced water deprivation on morphological traits, including root and shoot length, and total biomass, in all four studied genotypes. Water deprivation significantly reduced these traits, with more pronounced reductions observed at higher concentrations. However, spermine application improved these traits under water deprivation in all genotypes, possibly because spermine enhances antioxidant defenses, improves osmoregulation, and interacts with phytohormones and other stress-responsive molecules^30^. Similar results were recorded by Talaat et al.^44^, in maize plants, where foliar application of spermine elevated the adverse effects of water stress and significantly increased the plant growth and production.

## Conclusion

This study highlights the promising role of spermine as a bio-stimulant that can improve adverse effects of water deprivation in sweet corn genotypes, each exhibiting unique adaptive response. The role of spermine in mitigating oxidative stress, stabilizing photosynthetic pigments, and enhancing physiological functions during water deficit highlights its diverse contributions to resilience against water deprivation. The observed genotypic diversity spanning from moderate improvement across parameters in Dessert to Messenger’s emphasis on biochemical defenses, strong physiological and growth responses of Tyson, and balanced strategy of Royalty in water-use efficiency and biomass maintenance highlights that the effectiveness of spermine may differ based on genotype-specific characteristics.

The results indicate that integrating spermine treatment with careful genotype selection may provide an effective strategy for improving crop resilience in limited water environments. This method provides a route for sustainable sweet corn cultivation by integrating physiological and biochemical advantages with genetic traits, tackling the increasing difficulties brought about by climate change and water shortages. Future investigations may delve deeper into the application of spermine across various developmental stages and stress levels, combining molecular studies to unravel the regulatory pathways influenced by SPM with field trials to validate its practical efficacy under real-world agricultural conditions. This integrated approach would provide a comprehensive understanding of spermine’s potential in improving agricultural sustainability under water deficit conditions.

## Data Availability

The data generated and analyzed during this study is included in the article.

## Declarations

### Plant material

The collection of plant material complies with relevant institutional, national, and international guidelines and legislation.
